# Modulation of the Gut Microbiota to Control Antimicrobial Resistance (AMR)—A Narrative Review with a Focus on Faecal Microbiota Transplantation (FMT)

**DOI:** 10.3390/idr15030025

**Published:** 2023-05-09

**Authors:** Blair Merrick, Chrysi Sergaki, Lindsey Edwards, David L. Moyes, Michael Kertanegara, Désirée Prossomariti, Debbie L. Shawcross, Simon D. Goldenberg

**Affiliations:** 1 Centre for Clinical Infection and Diagnostics Research, Guy’s and St Thomas’ NHS Foundation Trust, King’s College, London SE1 7EH, UK; 2 Diagnostics R&D, Medicines and Healthcare Products Regulatory Agency (MHRA), Potters Bar EN6 3QG, UK; 3 School of Immunology and Microbial Sciences, Institute of Liver Studies, Faculty of Life Sciences and Medicine, King’s College, London SE1 1UL, UK; 4 Institute of Liver Studies, King’s College Hospital NHS Foundation Trust, London SE5 9RS, UK; 5 Centre for Host-Microbiome Interactions, Faculty of Dentistry, Oral and Craniofacial Sciences, King’s College, London SE1 1UK, UK

**Keywords:** microbiome, resistome, antimicrobial resistance, antimicrobial stewardship, gut microbiota, faecal microbiota transplantation, probiotics, bacteriophage, dysbiosis

## Abstract

Antimicrobial resistance (AMR) is one of the greatest challenges facing humanity, causing a substantial burden to the global healthcare system. AMR in Gram-negative organisms is particularly concerning due to a dramatic rise in infections caused by extended-spectrum beta-lactamase and carbapenemase-producing Enterobacterales (ESBL and CPE). These pathogens have limited treatment options and are associated with poor clinical outcomes, including high mortality rates. The microbiota of the gastrointestinal tract acts as a major reservoir of antibiotic resistance genes (the resistome), and the environment facilitates intra and inter-species transfer of mobile genetic elements carrying these resistance genes. As colonisation often precedes infection, strategies to manipulate the resistome to limit endogenous infections with AMR organisms, as well as prevent transmission to others, is a worthwhile pursuit. This narrative review presents existing evidence on how manipulation of the gut microbiota can be exploited to therapeutically restore colonisation resistance using a number of methods, including diet, probiotics, bacteriophages and faecal microbiota transplantation (FMT).

## 1. Introduction

Antimicrobials are designed to kill or attenuate the growth of microorganisms. Some microorganisms have evolved, or acquired from others, the ability to synthesise and secrete antimicrobial compounds into their local environment in order to outcompete other microorganisms. Some microorganisms have, in turn, evolved or acquired mechanisms to resist the activity of certain antimicrobials. Both production of, and resistance to, antimicrobials can be associated with a fitness cost. This has ensured the phenomenon has remained in relative equilibrium for aeons.

Since the discovery of penicillin in 1928 [1] and its first use in 1941 [2], antimicrobials have become intrinsic to medical practice, as well as in the food industry and agriculture. This has brought about numerous benefits: reduced mortality from sepsis, a life-threatening complication of infection; facilitation of advancements, such as organ transplantation, which would not have otherwise been possible; reduced food production costs through improved animal welfare and increased yields.

However, the sheer volume and often indiscriminate use of antimicrobials in recent decades has exerted a considerable selection pressure that has favoured the survival of microorganisms carrying antimicrobial resistance (AMR) and exacerbated the spread of resistance genes through microbial communities. Antimicrobial use was estimated to have increased by nearly 50% in humans between 2000 and 2018 [3] and is projected to rise by 67% in livestock by 2030 [4]. The concurrent explosion in global population density and mobility, widespread lack of access to adequate sanitation facilities and suboptimal infection control practices [5,6] have enabled resistant microorganisms to spread, facilitating another pandemic, AMR.

### 1.1. Antimicrobial Resistance (AMR)

Microorganisms can be intrinsically resistant to antimicrobials; for example, all Gram-negative bacteria are resistant to glycopeptides. Resistance can also be acquired, for example, on mobile genetic elements, e.g., a plasmid encoding for an antibiotic resistance gene (ARG) such as an extended-spectrum beta-lactamase (ESBL), or through changes to antibiotic targets, e.g., ciprofloxacin resistance due to mutations in the DNA gyrase, *gyrA*. Acquired AMR, particularly if transferable, is of greatest concern.

Infections secondary to antimicrobial-resistant microorganisms are more challenging to treat and are associated with worse clinical outcomes [7,8]. Evidence of their growing threat was highlighted in a recent Lancet report where bacterial AMR alone was estimated to be directly responsible for over 1.25 million deaths annually [9]. The World Health Organisation (WHO) predicts this could rise to 10 million by 2050. The gravity of the situation was emphasised by labelling AMR as one of the top 10 threats to global public health [10].

Several WHO priority pathogens (i.e., organisms deemed to be the most threatening) readily colonise the lower gastrointestinal tract, including carbapenem-resistant Enterobacterales (CRE), extended-spectrum beta-lactamase-producing Enterobacterales (ESBL-E) and vancomycin-resistant enterococci (VRE). Colonisation of the gut with these organisms often precedes infection at distant body sites. The pooled cumulative incidence of infection with resistant Gram-negative organisms in those who are colonised is estimated to be 14% [11]. Attempts to decolonise the GI tract of individuals carrying antimicrobial-resistant organisms (ARO) to prevent infection and reduce onward transmission using selective non-absorbable antimicrobials (selective digestive decontamination) have not proven effective [12] and may even promote AMR [13]. Therefore, alternative decolonisation strategies need consideration; one of these may be to modulate the gut microbiota.

### 1.2. The Gastrointestinal Tract as a Reservoir of AMR

The gastrointestinal (GI) tract has been known as a reservoir, and site of transferable ARG, since the 1950s [14]. Incubation of two bacterial cultures in vitro, one carrying ARG on a plasmid and another without, rapidly leads to the transfer of ARG to a proportion of previously antibiotic susceptible bacteria. Gram-negative bacteria, particularly Enterobacterales, frequently share ARG and are found in high concentrations in the lower gut. Gram-positive commensal species, such as Bacillota (previously Firmicutes), can also transfer resistance mechanisms between one another [15,16]. Transfer of ARG is noted to be higher in ecosystems with rich microbial abundance and diversity, such as the GI tract [17]. Thus, the gut is an ideal target to reduce the reservoir of AMR.

### 1.3. Colonisation Resistance

**Figure 1:**
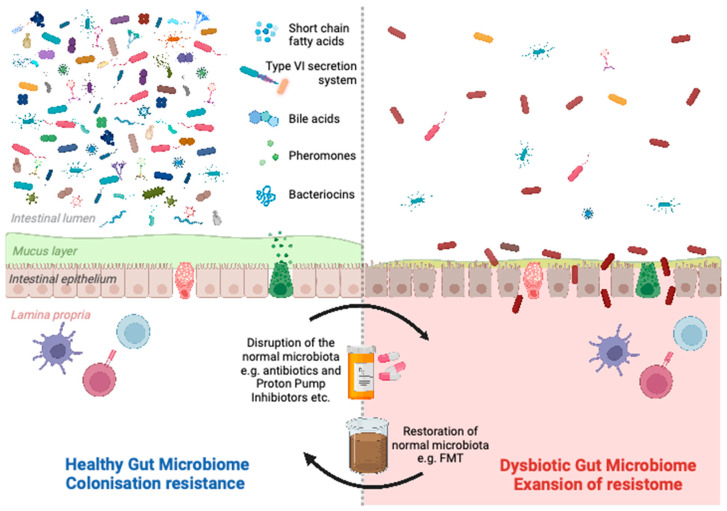
Mutualistic bacteria utilise amino acids and soluble carbohydrates as a source of energy, competing with potential pathogens. A layer of mucus, comprised of glycoproteins produced by goblet cells, lines the intestinal mucosa. This prevents potential pathogens from attaching to the intestinal epithelium by acting as a physical barrier. Many symbionts fortify the mucosal barrier, inhibiting pathogen outgrowth. Other bacteria can directly inhibit pathogens using the type VI secretion system (T6SS) or by secreting antimicrobial compounds such as bacteriocins, pheromone peptides and short-chain fatty acids (e.g., acetic, butyric and propionic acids). Others possess bile salt hydrolases that deconjugate bile acids or convert them from primary to secondary bile acids, which are able to inhibit the growth of pathogens. A range of symbionts induces the production of cytokines, such as IL-22 and IL-1β, which protect against colonisation by pathogens. Indigenous microbiota shape and modulate innate and adaptive immune responses. Interactions between bacteria and epithelial cells contribute to the regulation of epithelial permeability through the modulation of tight junctions. Diverse gut microbiota maintain optimal homeostasis but can be disrupted by antimicrobials and other drugs. This results in significant alterations in structure and function and permits the emergence of niches that pathogens can occupy.

Colonisation by indigenous microbiota is an effective barrier against the invasion of the gut by pathogens; this is known as colonisation resistance. Antibiotic-induced perturbations of the mouse gut microbiota dramatically increased susceptibility to infection by Salmonella through the loss of colonisation resistance [18]. There is a wide range of mechanisms by which the microbiota exert colonisation resistance, broadly separated into direct and indirect effects [19]. Bacteria can directly inhibit the growth of each other by competing for resources (spatial and nutritional/metabolic competition) or by producing a range of inhibitory compounds (antimicrobial peptides, e.g., bacteriocins, and metabolites, such as bile acids). In addition to microorganisms, the GI tract is abundant with bacteriophages and viruses targeting specific bacteria, resulting in lysis and death. Indirect mechanisms include the maintenance of the mucus barrier, limitation of oxygen, priming of innate immune defences, and induction of cytokines. The predominant mechanisms are summarised in Figure 1.

### 1.4. Modulation of the Gut Microbiota

The gut microbiota is dynamic, changing throughout life and in response to external factors, including diet, lifestyle habits such as exercise, or interventions, such as medications. Antimicrobial use, in addition to selecting for resistant microorganisms, can dramatically alter the structure and function of the gut microbiota and reduce colonisation resistance, permitting pathogen intrusion both in the immediate and longer-term [20,21]. Modulation of the gut microbiota to prevent colonisation occurring, eradicate ARO that have already colonised or reduce ARO numbers and/or prevent symptomatic infections caused by them, are all attractive endpoints with the potential to decrease the overall AMR burden.

This narrative review will focus principally on FMT as an intervention to achieve this, as it has the greatest body of literature. However, note that diet, prebiotics, probiotics or synbiotics (a combination of a pre- and probiotic) may have roles to play too. To date, one randomised controlled trial (RCT) has assessed the ability of FMT to eradicate colonisation with ARO [22], although others are in progress (Table 1). Other randomised studies have looked at AMR in post hoc analyses [23,24], and non-randomised studies have also looked at infection incidence with ARO post-FMT [25,26]. We will discuss ref. [22] in detail, followed by commentary on other studies.

## 2. Strategies to Reduce Expansion of the Gut Resistome by Modulating the Gut Microbiota

### 2.1. Diet/Prebiotics

Prebiotics are substrates selectively utilised by host microorganisms that confer a potential health benefit on the host. There is a great deal of overlap between dietary fibre and prebiotics, i.e., a substrate can fall under both categories, although not all prebiotics are fibres [28], making differentiation challenging. Observational data from a ‘healthy’ US cohort suggest a lower calorie, lower animal protein, higher fibre diet may reduce the abundance of ARG by promoting a colonic microenvironment favouring the presence of obligate over facultative anaerobes. Researchers could not control for potential confounders, such as previous antimicrobial exposure or assign causality, but the data do provide testable hypotheses for future intervention studies [29]. Propensity to infection with resistant GI tract organisms may also be influenced by diet: a higher intake of chicken was associated with cefotaxime resistance, and a higher intake of pork was associated with norfloxacin resistance in *E. coli* isolates in a Dutch cohort of elderly patients with urinary tract infections [30]. This suggests reducing or avoiding consumption of certain livestock could reduce AMR.

#### 2.1.1. Live Biotherapeutics

Probiotics are naturally occurring viable microorganisms that, when consumed in adequate amounts, may confer a health benefit on the host [31]. Studies of commercially available single and multi-strain probiotics, e.g., Yakult and SYMPROVE™, have demonstrated probiotics can reach the colon alive, colonise the mucosa and exert changes on the microbiome [32,33]. Through changes to the microbiota, as well as the associated metabolites, nutritional competition and secretion of antimicrobial compounds, probiotics may be able to inhibit the growth or expansion of resistant pathogens. In vitro and in vivo data demonstrate certain probiotics can reduce the abundance of Enterobacterales in the gut [34] and can have inhibitory activity against them [35,36], including ESBL-E [37]. A recent analysis of 14 clinical studies investigating the efficacy of probiotics to restore the microbiota in those with ARO (including nine studies targeting antimicrobial-resistant Enterobacterales) did not find any advantage of these products [38]. Many of the clinical studies investigating probiotics have used the eradication of ARO as the primary end-point (usually using culture-based techniques). Although the majority of probiotic studies have failed to meet this endpoint, it is possible that reducing the abundance of ARO may affect clinically beneficial outcomes in terms of reducing the incidence or severity of infections and other parameters (including quality of life outcomes) that have not been captured in these reports. An example of this is a randomised placebo-controlled trial of Vivomixx^®^, a nine-strain probiotic mixture given for 2 months in patients with gut carriage of ESBL [39]. Although more patients achieved successful decolonisation in the probiotic arm (12.5%) compared with the placebo arm (5%), this was not statistically significant. Although participants were followed up for 12 months, rates of clinical infection and other meaningful outcomes were not described. Engineered live biotherapeutics (eLBPs) are genetically modified microorganisms designed to perform specific therapeutic or diagnostic functions. Research into their use remains very much in its infancy [40], but there is theoretical potential to ‘engineer’ probiotics in order to maximise their benefit, for instance, to reduce the abundance of or to eradicate ARO.

#### 2.1.2. Bacteriophages

Bacteriophages are viruses that infect and replicate inside bacteria; they are ubiquitous and abundant in the environment. They have been used as therapeutics in former Soviet Union countries since the 1930s [41]. As more classes of antimicrobials were brought to market, the use of bacteriophages in Western countries dwindled. However, the antimicrobial resistance crisis has prompted renewed interest and a renaissance in phage therapy [42]. Most studies on the use of phages to target gut carriage of AROs are restricted to in vitro work or animal models [43,44,45,46]; however, there are a small number of case reports with successful outcomes. A carbapenemase-producing *Klebsiella pneumoniae* was successfully eradicated in a 57-year-old patient with Crohn’s disease and recurrent urinary tract infections requiring cystectomy and the creation of a ureterostomy with a ureteric stent [47]. A custom-made phage preparation was manufactured and administered orally and intra-rectally, which resulted in the eradication of the organism from stool, rectal swabs, urine and ureteric stent samples. A similar case in a 58-year-old renal transplant recipient with gut carriage and recurrent urinary tract infections with an ESBL-producing *Klebsiella pneumoniae* was also reported [48]. Following oral and intravesical administration of a customised phage, the patient did not suffer further UTIs; however, it is not clear if the *Klebsiella* was also eradicated from the GI tract. There is a lack of data from randomised controlled trials of phage therapy, which, together with the difficulty in obtaining suitable products and a lack of regulatory oversight, limits the wider application of this strategy at this current time [49].

#### 2.1.3. Faecal Microbiota Transplantation (FMT)

Another intervention specifically intended to modulate or replace the gut microbiota is faecal microbiota transplantation (FMT). This is the process of taking donated stool from screened healthy donors and processing it into a treatment administered to recipients for the purpose of improving their health. FMT is now well-established, and guidelines recommend treatment with FMT for patients suffering from recurrent *Clostridioides difficile* infection (rCDI), in whom the gut microbiota has become extensively disrupted or ‘dysbiotic’ [50,51]. FMT is postulated to work by reversing these perturbations and re-establishing a ‘healthy’ microbiota and metabolome that resists the outgrowth of *C. difficile* [52].

Interest in the ability of FMT to eradicate (or reduce) gastrointestinal (GI) carriage of ARO arose based on results in patients with rCDI, such as those reported by Millan et al. [53]. Stool samples of FMT donors and patients with rCDI prior to and following FMT were analysed. Donors had a similar burden of ARG in their global resistome compared to a ‘healthy’ control cohort (average of 3.4 vs. 6.0 ARG), mostly related to tetracycline resistance [54], whereas patients with rCDI had an average of >30 ARG. ARG was more diverse, encoding resistance to beta-lactams, fluoroquinolone and multidrug efflux pumps in patients with rCDI. FMT recipients who ‘responded’ to FMT, i.e., did not experience further CDI recurrence, were additionally noted to have a decrease in ARG carriage compared to non-responders. This reduction persisted for at least one year.

Similar results were seen in a post hoc analysis of the PUNCH CD study, which investigated RBX2660, a ‘microbiota restoration therapy’ akin to FMT, as a treatment for rCDI. Researchers found RBX2660 reduced the abundance of antibiotic-resistant Enterobacterales in recipients. Reduction in ARG carriage was proportional to the degree of donor microbiota engraftment [24]. In late 2022, this same product, now marketed as ‘REBYOTA™’, became the first licensed FMT product for the treatment of rCDI [55].

### 2.2. Randomised Controlled Trials (RCTs)

Huttner and colleagues undertook a four-centre open-label, publicly funded, randomised superiority study [22]. Adult participants with GI colonisation with either ESBL-E (*n* = 36) and/or carbapenamase-producing Enterobacterales (CPE) (*n* = 11) were randomised (1:1) to receive either a five-day course of an oral non-absorbable antibiotic combination followed by FMT, or no intervention. FMT was administered as either oral capsules, 15 ‘wet’ capsules on two occasions (*n* = 16), or liquid preparation (*n* = 6) via nasogastric (NG) tube. Each recipient received FMT from a single donor. The primary outcome was the intestinal carriage of ESBL-E/CPE in participants at 35–48 days following randomisation. The results of this study were inconclusive, although there was a trend towards benefit in the intervention arm that failed to reach statistical significance in the intention to treat analysis. There are a number of considerations when evaluating the study results.

Most notably, the target sample size was 64 participants based on an a priori power calculation. This was not achieved due to a delay in commencing study recruitment and an inability to extend the recruitment period due to funding restrictions. The primary outcome (decolonisation) was analysed using the intention to treat principle, with imputation (worst-case scenario) for missing data (9/22 in the FMT arm vs. 5/17 in the control arm, OR for decolonisation success 1.7). A per-protocol analysis was defined post-study but pre-data analysis and assessed 16 in the intervention group and 13 in the control group (8/16 in the FMT arm and 3/13 in the control arm, OR for decolonisation success 3.3). Despite a trend towards the benefit, neither analysis demonstrated statistical significance. Eligibility screening assessed >3000 individuals, although exact numbers and reasons for exclusion are not given. Where data were available, it would appear a large proportion of individuals were excluded due to their inability to follow-up, provide informed consent because they were immunosuppressed or they were unable to take study drugs. It is likely that many of the factors that increase the risk of individuals being colonised with ARO, e.g., multiple courses of antimicrobials, frequent hospital contact or prolonged hospitalisation, also make it more likely the individual is ineligible for recruitment. Under-recruitment, despite screening such a large number of individuals, highlights the difficulty of undertaking research in this area.

Although there is a rationale for administering antibiotics prior to FMT, i.e., to reduce the burden of ARO and create a niche for FMT engraftment, it does impact the ability to assess the efficacy of FMT alone. The lack of antimicrobial administration in the control arm compounds this. Antimicrobial administration to eradicate resistant pathogens has been evaluated previously, including by the authors of this study, with no evidence to suggest a benefit [56]. It may actually promote antimicrobial resistance, and in a later sub-study involving a cohort of individuals recruited to this trial, shotgun metagenomic analysis of stool post antibiotics, but prior to FMT, showed a statistically significant increase in ARG compared to baseline [13]. It is also plausible antimicrobials were not fully cleared from the gut lumen of recipients prior to FMT. For non-absorbable antibiotics, this would depend on the transit time of the participants: the protocol stated a washout period of only one calendar day without bowel lavage, which may not have been sufficient. The presence of residual antimicrobials could have impacted FMT engraftment.

FMT was administered in capsule formulation in two of the centres, as it was thought this would improve patient acceptability and facilitate recruitment. In the two remaining centres, it was administered as a liquid suspension due to the logistic and administrative difficulties of manufacturing capsules at these sites. Donations utilised to manufacture FMT were from donors screened as per local guidelines, which included screening for multidrug resistant organisms (MDRO). All material was processed in ambient air within two hours of donation. This production strategy is realistic and pragmatic, considering the time delay between stool production and delivery to a laboratory for processing, as well as the inherent difficulties in having access to the facilities necessary to manufacture under anaerobic conditions. Recruitment at sites using capsules was indeed higher (39 vs. 12); thus, it is plausible the recruitment target could have been reached were capsules used at all sites.

Further work is necessary to understand the differences in species abundances, viability and efficacy rates (outside of CDI) between liquid (fresh vs. frozen) and capsule (fresh vs. frozen vs. lyophilised) preparations. In this study, the majority of individuals received capsules. This delivery method will likely be the focus of future work in view of easier administration, reduced complication rates (as there is no need for endoscopy or enteral feeding tube) [57] and potentially greater patient acceptability [58].

Capsule FMT was administered over two consecutive days; each dose consisted of 15 capsules derived from 15–30 g of donor stool. FMT suspension was administered in a single aliquot of 80 mL derived from 40 g of donor stool. In both cases, the amount of raw stool used to manufacture the final product fell below the recommended (expert opinion) amount of ≥50 g [50] based on response rates in rCDI. Non-lyophilised products require a greater number of capsules to be administered, as by weight, the product is approximately 90–95% water. Lyophilisation can dramatically reduce the capsules required to administer the same amount of ‘product’ with minimal loss of bacterial viability [59]. The rationale for using <50 g of raw stool to manufacture FMT suspension is not clear unless this was to retain a similar dose to the capsule preparation. Whilst dosing is based on efficacy in CDI, it is plausible a beneficial effect of FMT was missed due to the under-dosing of participants.

FMT was manufactured from seven different donors, with each donor providing material to treat up to five participants. This is pragmatic and realistic of real-world practice. It is unlikely to be feasible to use only a single donor to ensure a more uniform product unless only a small number of recipients are recruited, which in turn would impact conclusions that could be drawn from study results. Recruitment of sufficient numbers of healthy donors can be a significant barrier to FMT research, with a broad range of reasons why potential donors are unwilling to participate and/or unsuitable to provide material [60,61,62].

Pooling donations or administering multiple FMT batches from different donors can help with standardisation across all study participants; however, this comes with the (theoretical) increased risk of transmitting a non-screened pathogen or disease trait. This risk must be balanced against any evidence of superiority. Synthetically produced products containing a range of bacterial strains are in development and will support greater uniformity between batches [63].

The study was open-label due to the practical barriers of masking the intervention. This is unlikely to have impacted the primary outcome data, as this was an objective measurement. The frequency of adverse events, including diarrhoea, was higher in the intervention group, which was expected in view of both the preceding antibiotics and FMT administration. However, reporting could have been subject to bias due to participant non-blinding. Participant loss to follow-up does not appear to have been significantly impacted by the frequency of adverse events, although one participant randomised to the control arm was lost to follow-up immediately after randomisation.

The Huttner et al. study recruited individuals who were either colonised with ESBL-E and/or CPE. Of these, the majority of the bacterial isolates were ESBL-producing *E. coli*. There are other bacterial species that may carry an ESBL or are capable of resisting carbapenems or producing carbapenemases that were not included, as well as other MDRO that colonise the GI tract, including VRE. FMT may have variable efficacy rates in the decolonisation of different MDRO; thus, care must be taken in generalising results to all resistant organisms.

### 2.3. Non-Randomised Trials

Shin and colleagues undertook a non-randomised controlled trial in multimorbid adult participants colonised with CRE or VRE over a two-year period in Korea [51]. Treatment allocation to FMT was assigned by participant preference. The study did not achieve the primary endpoint of MDRO decolonisation at one month post-FMT (26% in FMT arm vs. 10% in the control, *p* = 0.264). A number of analyses suggested a trend towards a reduction in MDRO carriage post-FMT in recipients, but only three-month decolonisation outcomes reached statistical significance. Additionally, the FMT group appeared to show reductions in the carriage of genera to which MDRO belonged, i.e., Enterobacterales or enterococci, and a more diverse microbiome compared to controls. The FMT group was younger than the control group, and although age was not found to be an independent risk factor for decolonisation in multivariate analyses, the impact of this difference on the overall result cannot be excluded.

The prospective cohort study conducted by Bar-Yoseph and colleagues recruited adult participants colonised with CPE in 2018–2019 [26]. Participants needed evidence of colonisation no more than one week prior to receipt of FMT and were not to have received antimicrobials for at least 48 h. In total, 15 of 39 eligible individuals went on to receive FMT, and 13/15 completed the two-day course, consisting of 30 capsules derived from an estimated 25–30 g of raw stool. Furthermore, 9/15 (60%) received FMT decolonised by one month. This is compared to 10/24 (42%) individuals in the control group. This difference was not statistically significant (*p* = 0.27). There were a number of clinical infections with CPE in the control group (9/24) vs. none (0/15) in the intervention group, as well as a higher mortality rate by the end of the study (8/24 vs. 0/15). The control cohort was recognised as being frailer and had spent longer in the hospital. Metagenomic analyses were performed on stool from eight ‘responders’ (CPE eradicated by one month) and five ‘failures’ (CPE persistence at one month) from the intervention group and compared to four donors. There were no analyses performed on individuals in the control cohort. Statistically significant differences in the composition of the microbiome were found in responders between their pre- and post-FMT samples, with post-FMT samples resembling donors and showing increased microbial diversity. These changes were not seen in ‘failures’. ‘Responders’ saw an increase in the number of species, including *Bifidobacterium bifidum*, a bacterium previously demonstrated to have anti-Enterobacterales activity [64]. There was also a decrease in the number of ARGs post-FMT. Taken together, the authors described taxonomic and functional shifts that they believed explained the mechanism of action of FMT in eradicating CPE. Whilst these data initially appear compelling, without comparison to a cohort of ‘responders’, i.e., those who eradicated CPE at one month but did not receive FMT, it is difficult to know which of these changes can be attributed to FMT and which may have occurred regardless.

As mentioned previously, and consistent with the Huttner study [22], 25–30 g is a relatively low amount of raw stool to use to manufacture FMT. This could potentially impact efficacy and may explain why results, although trending towards the benefit, did not reach statistical significance. There are promising data with respect to clinical infection caused by resistant organisms, with a statistically significant reduction in the intervention group compared to the control cohort (9/24 vs. 0/15, *p* = 0.007).

The cohort study by Ghani et al. undertaken during 2015–2019 focused on the prevention of MDRO infection rather than decolonisation in two high-risk groups: individuals in group 1 were haematology patients with planned immunosuppression, e.g., allogeneic haematopoietic stem cell transplant (allo-HSCT), and group 2 contained patients with recurrent MDRO-mediated invasive disease deemed clinically high-risk for further infections. These individuals suffered from recurrent urinary tract infections (UTIs) [25]. Group 1 patients were colonised with a range of ESBL-E, CRE and VRE; group 2 patients were predominantly colonised with ESBL-E. Study results demonstrated a significant reduction in the frequency of episodes of bacteraemia, length of hospital stay and carbapenem use in the intervention group compared to a comparator cohort made up of similar individuals managed over the same time period who did not receive FMT, either due to clinician or patient choice. Anaerobically prepared pre-frozen FMT was administered via a nasogastric tube to recipients. Each dose was manufactured from at least 50 g of donor stool and administered within 6 months of production. The decolonisation rate was modest by comparison (41%, 7/17 FMT recipients). It was not reported for the comparator cohort.

Battipaglia et al. also noted a lower-than-expected rate of infection in the post-allo-HSCT transplant period in individuals treated with FMT administered by enema or nasogastric tube [65]. The larger dose of FMT (50–100 g raw stool) compared to some other studies seemed to be well tolerated, with only self-limiting side effects reported, some of which may have been related to bowel preparation. It should also be noted that the FMT was anaerobically prepared, which could have implications on composition and potentially for efficacy, and that this was a highly selected population of individuals, thus limiting the broader applicability of results. The spontaneous decolonisation rate in the comparator cohort is not provided.

A post hoc analysis of two RCTs recruiting, in total, 40 patients with end-stage cirrhosis assessed the impact of FMT on ARG abundance in the stool using the Comprehensive Antibiotic Resistance Database (CARD) [23]. Following capsule FMT there was evidence of a reduction in ARG associated with both Gram-positive (e.g. VanH) and Gram-negative bacteria (e.g., Oxy beta-lactamase). There was also a reduction in vancomycin resistance genes (VanW) in the enema FMT group. Interpretation of other results is complicated by pre-procedure use of antibiotics, which in turn was associated with a transient increase in certain ARGs, such as beta-lactamases and those associated with quinolone resistance. These changes could not be attributed to donors, and the increase in ARG was not completely reduced by FMT. Limitations on the broader applicability of the study results are the specific population recruited, that participants were receiving regular antibiotics, lactulose and a proton pump inhibitor, the delivery of antibiotics prior to liquid enema FMT and that the authors only evaluated for ARG, i.e., did not attribute them or assign them to specific bacterial species. Additionally, a relatively small amount of raw stool (15 wet capsules containing a total of 4.125 g) was used.

Several other groups demonstrated a potential reduction in VRE carriage following FMT. Eysenbach and colleagues compared 16 individuals with rCDI and concurrent VRE carriage following donor (*n* = 9) or, as a control, autologous (*n* = 7) FMT recruited across six hospital sites [66]. At the first time point measured, all nine individuals who received donor FMT had decolonised (VanA not detectable on PCR-based testing) vs. 3/7 in the control group. However, by the time of the final follow-up, 6/7 in the control group had spontaneously decolonised. With such high rates of spontaneous decolonisation, it emphasises the importance of control groups and adequately powered studies and also questions the necessity of an intervention to ‘treat’ VRE colonisation. Davido et al. [67] and Dinh et al. [68] also demonstrated high rates of decolonisation (>80%) in FMT recipients. However, with no control group for comparison, and evidence that spontaneous decolonisation occurs frequently, attribution to the FMT alone cannot be made.

Saïdani and colleagues described a high rate (80%) of decolonisation of CPE or CP-Acinetobacter two weeks after fresh FMT via a nasogastric tube in a cohort of individuals whose ongoing care was impacted by colonisation with ARO, compared to 10% in a comparator cohort [69]. FMT recipients had a reduction in time to hospital discharge compared to controls. Cases were heavily pre-treated prior to FMT, including a three-day nasopharyngeal decolonisation protocol, two bowel lavages at Day(D)-5 and D-1 prior to FMT and a five-day course of non-absorbable antibiotics. Additionally, all indwelling lines, including gastrostomies and urinary catheters, were replaced shortly before the administration of FMT. Whilst there is the justification given for the intensive regimen the cases were put through, it is likely to limit the broader applicability of these results, as compliance with such a protocol is likely to be limited and, if scaled up, may itself be associated with significant complications. It is also not possible to assess the impact of FMT alone, in view of the number of interventions they received.

Lee et al. evaluated the gut microbiome before and after up to three doses of FMT in 10 KPC-CPE carriers with risk factors for prolonged colonisation, e.g., continued carbapenem use, concurrent CDI or long duration of hospitalisation [70]. Five were decolonised by 51 days after one dose of FMT, and four subsequently decolonised within 34 days of a second or third FMT. One participant who received only one dose failed to decolonise by the time of the final follow-up (138 days). Recipients were split into two categories: early decolonisation carriers (EDCs) who decolonised after one FMT and late decolonisation carriers (LDCs) who decolonised after two or more doses. They noted that EDCs increased bacterial diversity vs. LDCs, although not statistically significant, and were less dissimilar to donors. There was the suggestion that pre-existing microbiota may have a role to play in the efficacy of eradicating ARO. EDCs had a higher relative abundance of Bacteroidota and Bacillota and increased overall diversity compared to LDCs pre-FMT. These differences were not statistically significant but are nevertheless of interest and worthy of further evaluation. The authors suggest there may be a donor-dependent effect on decolonisation, although none of the differences between donors reaches statistical significance, likely due to the small sample size.

Seong and colleagues recruited 30 adult and 5 paediatric participants colonised with CRE and/or VRE [71]. Forty-eight individuals who fulfilled eligibility criteria, but declined to participate, were assessed retrospectively as a comparator cohort. Decolonisation was achieved for 15/35 (42.9%) of the participants at one-month post-FMT and for 24/35 (68.6%) of the participants by the end of follow-up in the intervention group, compared to 13/48 (27.1%) in the control group. In total, 9/24 (37.5%) of the decolonised participants experienced recolonization, presumably with the same bacterium (relapse rather than reinfection). No breakdown is given for decolonisation rates for adult and paediatric participants. This is potentially of relevance as the microbiota in children has been demonstrated to be more dynamic. However, they do not report an impact of age on decolonisation efficacy in a multivariate analysis. For participants colonised with VRE, increased richness and diversity of gut microbiome prior to FMT were associated with a higher rate of decolonisation. Participants who decolonised within 14 days of FMT had significantly lower abundances of Verrucomicrobiota and Pseudomonadota compared to those who did not. They also identified several species that were present in early decolonisers, such as *Clostridium ramosum*, that were not present in other participants.

Both of these studies suggest pre-existing microbiota could predict the likelihood of efficacy of FMT to eradicate ARO; the more disordered the microbiota, or the more ‘dysbiotic’ an individual’s gut microbiota is, the more difficult it is to restore and presumably reach a state of colonisation resistance to ARO. Repeat FMT may gradually reverse dysbiosis and ultimately facilitate decolonisation in those who fail to decolonise after one or more doses of FMT.

Bilinski et al. treated haemato-oncology patients with liquid FMT administered via the upper GI route [72]. GI colonisation with ARO has been associated with inferior outcomes in patients undergoing allo-HSCT. They recruited 20 participants colonised with a range of Gram-negative MDRO who received a total of 25 FMTs. Their primary endpoint of decolonisation at one month was achieved in 60% of FMTs (15/25) and was more likely if individuals did not receive antibiotics in the week following FMT delivery (79% vs. 36%, *p* ≤ 0.05). Decolonisation for individuals colonised with NDM-1 *K. pneumoniae* was more likely if the FMT contained a higher number of operational taxonomic units (OTUs), i.e., was more ‘diverse’, and there was a greater abundance of *Barnesiella* spp., *Bacteroides* and *Butyricimonas*. This implies that the efficacy of FMT may be impaired by antibiotics administered shortly after FMT and that the composition of FMT may affect outcomes. Reassuringly, there were no safety signals towards FMT, with no severe adverse events. Battipaglia and colleagues also demonstrated safety in administering FMT to patients with haematological disorders, but again, all but one had a neutrophil count >1.0 at the time of FMT. Result interpretation is limited by the absence of a comparator group, although they reference historical literature, which points to low rates of, and long median time to, spontaneous decolonisation in this patient group [65].

## 3. Conclusions

Presently, there is low-quality evidence supporting the hypothesis that modulation of the microbiota (using FMT and/or other strategies) results in a reduction in MDRO and/or ARG GI tract carriage. For FMT specifically, as an intervention, the non-randomised nature of most studies, small sample sizes, heterogeneity in FMT delivery methods, antimicrobial use pre- and post-FMT, ‘dose’ of FMT used, variability in the FMT product, different MDRO targets and non-standardised criteria used to assess decolonisation, make it difficult to draw firm conclusions regarding efficacy, and no routine recommendation can be made about the use of FMT for this indication at present. Adequately powered, well-designed, multi-centre RCTs are necessary to definitively address this question. However, these are challenging to conduct and expensive, so researchers should learn from other’s experiences. There is even less evidence to support the use of specific dietary interventions, prebiotics, live biotherapeutics and bacteriophages, but there is potential for one or more of these to play a role in the future.

A number of randomised studies investigating the effect of FMT on colonisation and/or infection with MDRO are registered as being in set-up, are currently recruiting or have been recently completed with results being awaited (Table 1). If these can recruit to target, the outcomes generated should provide a much clearer picture of the situation. Perhaps most encouraging from the data is the suggestion that FMT may reduce the incidence of symptomatic infections with MDRO. Although decolonisation is often the intended outcome, this result is likely to hold greater clinical significance, with the potential to reduce the usage of last-line antibiotics, as well as reduce patient morbidity and mortality. We suggest this is considered as an outcome measure in all future studies.

## Figures and Tables

**Table 1:** A list of actively recruiting, or recently completed, randomised studies investigating FMT as a treatment for the eradication of gastrointestinal carriage of resistant organisms. Adapted from ref. [27].

NCT/EUCTR Number	Study Design and Location	Enrolment (*n*)	Start Date	Estimated Completion (and Preliminary Results If Posted)	Inclusion Criteria	Arms and Interventions	Primary Outcome (Secondary Outcome Is Mentioned If Relevant)
NCT05632315	Randomised, open-label, controlled trial **USA**	150 estimated	January 2023	January 2026	Adults with MDRO infection receiving appropriate antimicrobial therapy for at least 5 days, with at least 2, but no more than 7 days of treatment remaining	**Group 1:** FMT via enema or suspension **Group 2:** standard of care	Decolonisation rate at 1 month. Frequency of adverse events in 6 months
NCT03802461	Randomised, open-label, controlled trial **Canada**	40 estimated	March 2019	March 2024	Adults with ≥1 rectal swab, groin, stool, or urine specimen positive for CRE within the past month	**Group 1:** bowel lavage followed by FMT (50 g healthy donor stool)administered by enema, given on 3 occasions **Group 2:** no intervention	Decolonisation rate (undefined) after 3 months
NCT04188743	Randomised, double-blind, controlled trial **Belgium**	150 estimated	December 2019	December 2023	Adults with at least two consecutive confirmations of MDRO colonisation in faeces	**Group 1:** allogenic FMT: 50 g of healthy donor stool, frozen, administered by nasoduodenal tube **Group 2:** autologous FMT: 50 g of own stool, frozen, administered by nasoduodenal tube **Group 3:** no intervention	Decolonisation rate, defined as three consecutive negative stool cultures in minimal time span of 2 weeks, after 1 month after treatment
NCT04181112	Randomised, open-label, controlled trial **Canada**	90 estimated	November 2019	November 2023	Adult renal transplant recipients colonised with a multidrug-resistant organism (undefined), confirmed by rectal swab or stool culture	**Group 1:** FMT using retention enema **Group 2:** antibiotic pre-treatment (undefined) followed by FMT using retention enema **Group 3:** no intervention	Decolonisation rate, defined by negative culture/PCR at 14 and 30 days post-FMT
NCT04746222	Randomised, double-blind, controlled trial **Singapore**	108 estimated	July 2021	July 2023	Adults (age ≥21) colonisation with CRE, confirmed with at least one positive rectal swab (PCR) taken ≤7 days before randomisation. Antibiotics ceased for at least 48 h pre-randomisation evaluation.	**Group 1:** single dose of 30 oral capsules containing healthy donor stool **Group 2:** single dose of 30 placebo capsules	Decolonisation rate, defined by negative rectal swab (PCR/culture), at 12 weeks
NCT04759001	Randomised, double-blind, controlled trial **Italy**	52 estimated	February 2021	February 2023	Adults with CRE colonisation, confirmed by a rectal swab	**Group 1:** FMT by colonoscopy with healthy donor stool **Group 2:** placebo (water) administered through colonoscopy	Decolonisation rate, defined by negative rectal swab at 4 weeks
NCT04431934	Randomised, open-label, controlled trial **Spain**	437 estimated	November 2020	December 2022 (still recruiting January 2023)	Adults with documented rectal colonisation with multidrug-resistant Gram-negative bacteria, eligible for routine digestive decolonisation	7 days of non-absorbable antibiotics followed by: **Group 1:** FMT 2 doses, once a week, 14–17 capsules per dose (dose is equivalent to 50 g of healthy donor stool) **Group 2:** 2 sachets of probiotics every 12 h for 14 days **Group 3:** no intervention	Decolonisation rate, defined as negative rectal swab after 60 days
NCT04760665	Randomised, double-blind, controlled trial **Spain**	120 estimated	April 2021	July 2022 (still recruiting January 2023)	Adult patients colonised with KPC-producing *Klebsiella pneumoniae* (undefined) without an active infection in the month prior to inclusion	**Group 1:** four oral capsules containing healthy donor faeces **Group 2:** four oral placebo capsules	Decolonisation rate (undefined) at 30 days
NCT04146337	Randomised, open-label, controlled trial **Israel**	3/60 actual	October 2020	June 2022 (marked as completed, no results published yet)	Adult inpatients positive for CRE of any strain and resistance mechanism in rectal surveillance stool samples, with or without CRE clinical samples. A positive rectal swab within one week before randomization is mandatory.	**Group 1:** FMT, 15 capsules a day for two consecutive days after an eight-hour fast **Group 2:** no intervention	Decolonisation rate, defined as three consecutive negative rectal cultures, at 28 days
EUCTR2019-001618-41	Randomised, participant-blinded, controlled, feasibility trial **UK**	44/80 actual	September 2019	March 2022 (follow-up to complete June 2023)	Adults with documented gastrointestinal carriage of ESBL-E or CRE (stool sample) in the 21 days prior to consent and symptomatic infection with the target organism in the preceding 6 months	**Group 1:** FMT capsules (80 g of healthy donor faeces per 5 capsules) on three consecutive days. Pre-treatment with a proton-pump inhibitor **Group 2:** placebo capsules	To determine the feasibility and acceptability of administering encapsulated FMT to participants colonised with ESBL-E/CPE. A secondary objective is to provide early evidence of efficacy (decolonisation rate by culture/PCR at days 10, 40, 100 and 190).
NCT03063437	Randomised, double-blind, controlled trial **USA**	9/? actual	August 2017	February 2019 **Preliminary results:** VRE decolonisation at day 10: 1 out of 4 participants in the FMT group and 1 out of 5 participants in the placebo group	Adults colonised with VRE (by stool culture) in the last 14 days	**Group 1:** single dose of FMT (30 capsules per dose) **Group 2:** placebo capsules	VRE decolonisation rate (absence of VRE on stool culture) at day 10
NCT03061097	Randomised, double-blind, controlled trial **USA**	4/20 actual	July 2017	June 2019 **Preliminary results:** 0 out of 4 patients were decolonised 28 days after autologous FMT	Long-term care residents with a history of an infection requiring antimicrobial treatment at the discretion of the treating physician	**Group 1:** Autologous 125 mL FMT (biobanked stool from the same patient collected before infection requiring antibiotics) via enema **Group 2:** Placebo	Safety (short-term) at Day 7 is defined as NIH Grade ≥2 adverse events. Secondary objective: among patients with MDRO colonisation at day 0: decolonisation rate at day 3, day 7 and day 28
EUCTR2019-004402-10-FR	Randomised, double-blind, controlled trial **France**	214 estimated	Not mentioned	Not mentioned (marked as ongoing)	Adult patients colonised with ESBL-E or CRE, assessed with stool culture and having suffered from infection with ESBL-E in the previous 12 months	**Group 1:** FMT capsules (*n* = 25) for two days in a row **Group 2:** placebo	Decolonisation rate at 30 days, determined by (undefined) culture methods

## Data Availability

No new data were created.
